# Twins Singularly Connected, &c., &c.

**Published:** 1850-07

**Authors:** Napoleon B. Anderson

**Affiliations:** Louisville, Kentucky


Art. IV.—*Twins Singularly Connected, &c., &c.* By Napoleon B. Anderson, M. D., of Louisville, Kentucky.

About 10 o'clock, A. M., on the morning of Tuesday, the 21st of May last, I was called to see Mrs. M., aged nineteen years, supposed at the time, to be in labor with her first child. I found her composed, free from fever, with occasional pains of short duration. On ascertaining that she had carried her child only seven months, and over-exerted herself on the previous day, I was fearful of a premature labor. On examination per vagina, the os uteri was found dilated to the size of a small goose quill, with slight hemorrhage from the vagina, which was supposed to have been caused by reaching up and scrubbing some windows. For the purpose of checking the hemorrhage, cold cloths were applied to the vulva and pubes, and one of the following pills given every hour:

℞. Extract hyoscyamous, grs. x.
Plumbi acetatis, grs. xxv.
Pulv. opii, grs. iv.
Oil menth, pip, gutt., ij.
Misce. ft. Pilulæs, xv.

At 9 P. M., the pains and flooding had ceased, and the patient remained free from them until the 22d, at 11 o'clock, P. M., when I was sent for again. I found the pains stronger and more frequent, than at first, with an absence of flooding, and the os uteri, was as large as a five cent piece. Thinking it prudent to interfere no farther, she was allowed nothing but a pill occasionally, until the 23d, at 10 o'clock, A. M., when the os uteri had dilated larger than a dollar, and flooding commenced again, with pains too feeble to expel the child. Twenty grains of ergot were ordered in three equal portions; one to be given every half hour in warm water. After the exhibition of the third powder, the patient gave birth to twins, male and female; one a head, and the other a foot presentation. The placenta and child were born with one pain. No flooding of consequence followed, and in ten days the mother was well.

On an examination of the children, (for they died in twenty minutes after birth,) they were found attached to each other at the umbilicus, by a cartilaginous, cylindrical tube, which was enclosed within a sac, formed by a continuation of the skin from the abdomen of both children, passing off gradually in a funnel shape, from each child about two and a half inches long, and an inch in diameter, from the center of which arose the placental chord of usual size, with a medium placenta. On laying open the tube, it was found to contain two intestines, of the size of the cecum, one going to the male from the female, and vice versa. The children were well proportioned, and may have other peculiarities about them, besides those detailed. They have not, as yet, been fully examined. The writer has them in his possession.

